# Working with patients affected by macular eye conditions

**Published:** 2025-01-31

**Authors:** Raja Narayanan

**Affiliations:** 1 Director: Anant Bajaj Retina Institute, LV Prasad Eye Institute, Hyderabad, India.

**Age-related macular degeneration (AMD) and diabetic macular oedema (DMO) are serious conditions, often leading to loss of vision. AMD and DMO affect the central vision, making it hard to read, recognise faces, and see objects clearly. Anti-VEGF drugs can help to stabilise both conditions and slow down vision loss.**

**Figure:**
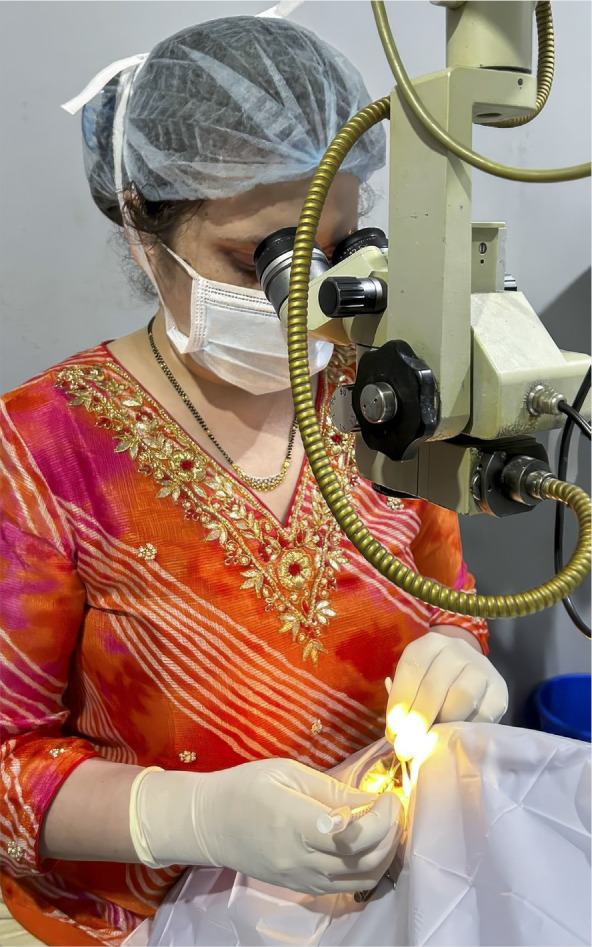
Giving an anti-VEGF injection. INDIA

© RAJA NARAYANAN CC BY-NC-SA 4.0

The prevalence of AMD is estimated to be 170 million globally, with 90% of patients affected by dry AMD.^1^ It is estimated that the number of people with diabetes mellitus (DM) worldwide will increase by 46%, from 537 million in 2021 to 783 million in 2045.^2^

The most common treatment for wet AMD and DMO is anti-vascular endothelial growth factor (anti-VEGF) injections, given into the eye. Intravitreal injections of anti-VEGF drugs can help to stabilise the disease, but the treatment requires frequent visits to the doctor and repeated injections, over many years. This can be challenging and stressful for patients and their families.

Financial costs, anxiety, and access to care are major barriers that can prevent patients from sticking to their treatment plan.^3^ Undertreatment is the most common cause for poor visual outcomes.

“Financial costs, anxiety, and access to care are major barriers that can prevent patients from sticking to their treatment plan.”

Studies from various countries show that continued treatment over many years is required in AMD and DMO, and that most patients regain some vision over time (an average of 10 letters). While visual improvement may be limited, the treatment prevents the severe vision loss that would otherwise be inevitable.

## Understanding patients’ concerns

To improve adherence to treatment, it is vital to understand patients’ concerns beyond just the cost. Factors like anxiety, lack of information, and family burden can prevent adherence to treatment. Improving patients’ understanding of the condition, treatment routine, and the possible treatment outcomes, can make them more likely to continue with their treatment.

A common concern among patients receiving an injection for the first time is that it will be very painful. I explain to them that we perform the injection after instilling anaesthetic eye drops into the eye, and there will be a pricking sensation rather than pain. Patients who undergo repeat injections are usually not anxious about the pain.

A bigger concern among patients is the visual outcome after multiple injections. Even though patients are counselled about the possibility of only limited improvement with this treatment, their expectations can be high. It is important to talk to family members as well, and keep their expectations reasonable. Explain to them that the main goal of the treatment is to maintain vision, and their eyesight is likely to deteriorate if they do not follow the schedule of injections as prescribed.

Most patients with DMO and AMD will experience some visual impairment and will benefit from referral to low vision and rehabilitation services.
